# Cardiovascular Disease and Chronic Pulmonary Disease Increase the Risk of Short-Term Major Postoperative Complications after Robotic-Assisted Radical Prostatectomy

**DOI:** 10.3390/medicina60010173

**Published:** 2024-01-18

**Authors:** Carolin Siech, Antonia Gruber, Mike Wenzel, Clara Humke, Pierre I. Karakiewicz, Luis A. Kluth, Felix K. H. Chun, Benedikt Hoeh, Philipp Mandel

**Affiliations:** 1 Goethe University Frankfurt, University Hospital, Department of Urology, 60629 Frankfurt am Main, Germany; 2 Cancer Prognostics and Health Outcomes Unit, Division of Urology, University of Montréal Health Center, Montréal, QC H2X3E4, Canada

**Keywords:** comorbidities, Clavien Dindo classification, postoperative complications, radical prostatectomy, prostate cancer

## Abstract

*Background and objectives*: Certain comorbidities may be associated with a higher risk of complications after robotic-assisted radical prostatectomy. *Material and Methods*: Relying on a tertiary care database, we identified robotic-assisted radical prostatectomy patients (January 2014–March 2023). Short-term major postoperative complications were defined according to Clavien Dindo as ≥IIIa within 30 days after robotic-assisted radical prostatectomy. *Results*: Of 1148 patients, the rates of postoperative Clavien Dindo IIIa, Clavien Dindo IIIb, Clavien Dindo IVa, and Clavien Dindo IVb complications were 3.3%, 1.4%, 0.3%, and 0.2%, respectively. Of those, 28 (47%) had lymphoceles, and 8 (13%) had bleeding-associated complications. Patients with cardiovascular disease (8 vs. 4%) or chronic pulmonary disease (13 vs. 5%) were more likely to have complications. In multivariable logistic regression models, cardiovascular disease (odds ratio: 1.78; *p* = 0.046) and chronic pulmonary disease (odds ratio: 3.29; *p* = 0.007) remained associated with an increased risk of postoperative complications. *Conclusions*: Complications after robotic-assisted radical prostatectomy are predominantly manageable without anesthesia. Concomitant cardiovascular disease and chronic pulmonary disease were both associated with a higher risk of postoperative complications.

## 1. Introduction

Prostate cancer is one of the most prevalent cancers among men [1]. For patients at the localized stage of disease, radical prostatectomy represents a well-established curative treatment option, which is recommended by national and international guidelines [2,3]. Recently, minimally invasive surgical approaches, such as robotic-assisted radical prostatectomy (RARP), have gained in importance alongside open radical prostatectomies and are being used more and more frequently to treat patients with prostate cancer [4,5]. Nevertheless, RARP, like open radical prostatectomy, is still associated with a certain risk of short- and long-term postoperative complications [6]. Postoperative complications after RARP may not just impact patients’ well-being but also prolong inpatient stays [7,8]. In addition to intraoperative factors, such as estimated blood loss, as well as pelvic lymph node dissection (PLND) [9], preoperative factors, such as a patient’s age; American Society of Anesthesiologists (ASA)’s physical status; body mass index (BMI); lymph node invasion; and neoadjuvant therapy, are likely to affect the rates of postoperative complications after radical prostatectomy [8,10,11,12,13,14]. In an aging society, comorbidities and a history of complex medical conditions are of major importance in cancer and non-cancer patients [15]. Since surgical and medical co-management as well as preoperative optimization of comorbidities can improve and advance the surgical outcomes of radical prostatectomy, it is indispensable to identify those patients with localized prostate cancer who are at a higher risk of short-term major postoperative complications after RARP [16]. 

In historical series of patients with prostate cancer who underwent radical prostatectomy before 2014, patients with certain comorbidities, such as diabetes mellitus, chronic kidney disease, atrial fibrillation, previous coronary intervention, or previous cerebrovascular attack, were at a significantly higher risk of postoperative complications [11,12,17,18]. Due to new insights into the prostate anatomy [19,20,21] and significant advancements in surgical techniques over time [22,23], these results may not be applicable to more contemporary cohorts of patients with prostate cancer. Therefore, the contemporary short-term major postoperative complication rates after RARP in patients with versus without the most prevalent chronic diseases are unknown. No previous study has comprehensively assessed the association between the most prevalent chronic diseases and short-term major postoperative complications after RARP, using the same inclusion and exclusion criteria for the study cohort selection. 

Based on this knowledge gap, we hypothesized that the short-term major postoperative complications after RARP do not differ significantly between prostate cancer patients with the most prevalent comorbidities, such as arterial hypertonia, elevated blood fat levels, diabetes mellitus, cardiovascular disease, chronic pulmonary disease, chronic kidney disease, other cancer, mental disorder, and neurologic disease, relative to their counterparts without the respective comorbidity. To address this hypothesis, we relied on the most contemporary cohort of patients with localized prostate cancer and who underwent RARP in a tertiary care referral center between January 2014 and March 2023.

## 2. Materials and Methods

### 2.1. Study Population

Relying on our prospectively maintained institutional tertiary care database, we retrospectively identified patients with prostate cancer who underwent RARP between January 2014 and March 2023. Eleven moderately to highly experienced surgeons performed RARP with or without pelvic lymph node dissection using a transperitoneal approach. In November 2017, full functional-length urethral sphincter preservation (FFLU) and neurovascular structure-adjacent frozen-section examination (NeuroSAFE) were implemented as the standard of surgical technique in our tertiary care referral center [16]. The FFLU technique relies on identifying and precisely separating the striated and smooth muscle layers of the urethral sphincter, reaching from the prostate apex to the colliculus of the prostate. In the NeuroSAFE technique, the preservation of the neurovascular bundle relies on the technique of an intraoperative frozen section. If positive surgical margins in the region, where the neurovascular bundle was resected, are detected in the intraoperative frozen section, it used to be the standard procedure to also perform a secondary resection of the implicated site of the neurovascular bundle. The indication for RARP was histologically confirmed adenocarcinoma of the prostate. All patients gave their informed written consent to participate in this study. Ethical approval by the local ethics committee of the University Hospital Frankfurt has been obtained prior to data collection. The reporting has been reviewed in accordance with the precepts established by the Helsinki Declaration.

### 2.2. Definition of Variables for Analyses

Major postoperative complications after RARP were prospectively recorded in the electronic medical records of each patient, and retrospectively collected for the current study. The severity of complications was graded according to the Clavien Dindo classification [24]. As the primary endpoint, short-term major postoperative complications were classified as Clavien Dindo ≥ IIIa within 30 days after RARP. Concomitant comorbidities were assessed during preoperative medical history interviews by both experienced anesthesiologists and urologists. Subsequently, the short-term major postoperative complications were grouped into categories, namely arterial hypertonia, elevated blood fat levels, diabetes mellitus, cardiovascular disease, chronic pulmonary disease, chronic kidney disease, other cancer, mental disorder, and neurologic disease, based on previous publications as defined in Supplementary Table S1 [25,26,27,28,29]. Furthermore, obesity was defined as a BMI greater than or equal to 30. Moreover, we also assessed the intake of platelet aggregation inhibitor and intake of anticoagulant medication, as well as the history of previous urological and/or abdominal surgery. The covariates consisted of age (years, continuously coded), D’Amico risk group (low vs. intermediate vs. high), Gleason sum score at biopsy (6 vs. 7 vs. ≥8), preoperative clinical tumor stage (≤cT2a vs. cT2b vs. ≥cT2c vs. unknown), initial prostate specific antigen (PSA) value (ng/mL, continuously coded), neoadjuvant systemic therapy (yes vs. no), surgeon volume (continuously coded), surgical technique (FFLU + NeuroSAFE vs. standard), pelvic lymph node dissection (yes vs. no), and pathologic lymph node stage (N0 vs. N1 vs. NX).

### 2.3. Statistical Analyses

The baseline characteristics and rates of short-term major postoperative complications were tabulated. The descriptive statistics included medians and interquartile ranges (IQR) for continuously coded variables and frequencies and proportions for categorical variables. To assess the distribution, the non-parametric Wilcoxon rank sum test evaluated the statistical significance of medians’ differences for categorical variables. Pearson’s Chi-squared test examined the statistical significance in proportions’ differences for categorical variables and Fisher’s exact test was used to compute an exact *p* value when expected counts were less than 10. Exploratory univariable analyses relying on logistic regression were performed to address the association between the most prevalent comorbidities and short-term major postoperative complications (Clavien Dindo ≥ IIIa). Odds ratios with 95% confidence intervals were calculated. Only comorbidities with a *p* value < 0.05 in the univariable analyses were eligible to be included in the multivariable logistic regression models. The adjustment variables consisted of tumor and institutional characteristics.

The R (software environment was used for statistical computing and graphics (R version 4.2.2; R Foundation for Statistical Computing, Vienna, Austria). All the tests were two-sided, with a level of significance set at *p* < 0.05 [30].

## 3. Results

### 3.1. Descriptive Characteristics

Within our institutional tertiary care database, we identified 1148 patients who underwent RARP between January 2014 and March 2023 (Table 1). Overall, short-term major postoperative complications (Clavien Dindo ≥ IIIa) occurred in 60 (5%) patients. According to the Clavien Dindo classification, the rates of postoperative Clavien Dindo IIIa, Clavien Dindo IIIb, Clavien Dindo IVa, and Clavien Dindo IVb complications were 3.3%, 1.4%, 0.3%, and 0.2%, respectively. No Clavien Dindo V postoperative complication occurred. Of those patients with short-term major postoperative complications (Clavien Dindo ≥ IIIa), 28 (47%) patients had lymphoceles, 8 (13%) patients had bleeding-associated complications, 5 (8%) patients had bowel-associated complications, 3 (5%) patients had infections, 3 (5%) patients had fascia or wound dehiscence, 2 (3%) patients had cardiac complications, 1 (2%) patient had thrombo-embolic complications, and 10 (17%) patients had other complications (e.g., urinary retention, compartment syndrome, and ureter damage).

The patients treated with RARP who experienced short-term major postoperative complications (Clavien Dindo ≥ IIIa) more frequently exhibited high-risk prostate cancer (47 vs. 26%; *p* = 0.002), more frequently had a Gleason score ≥ 8 (33 vs. 19%; *p* = 0.02), had a higher mean PSA value (9.7 vs. 7.0 ng/mL; *p* = 0.002), and more frequently exhibited lymph node invasion (20 vs. 6%; *p* < 0.001; Table 2). Moreover, RARP-treated patients with short-term major postoperative complications received more often neoadjuvant systemic therapy (17 vs. 5%; *p* = 0.002) or pelvic lymph node dissection (97 vs. 88%; *p* = 0.04) compared to patients without short-term major postoperative complications. Conversely, no differences between the two groups were observed according to age at surgery, preoperative clinical tumor stage (cTstage), surgeon volume, operation time, or surgical technique. 

In general, 486 (42%) patients were preoperatively diagnosed with concomitant arterial hypertonia, followed by 269 (23%) patients with cardiovascular disease, 237 (21%) patients with obesity, 107 (9%) patients with other cancer, 105 (9%) patients with diabetes mellitus, 100 (9%) patients with neurological disease, 64 (6%) patients with elevated blood fat levels, 54 (5%) patients with chronic pulmonary disease, 32 (3%) patients with chronic kidney disease, and 31 (3%) patients with mental disorders (Table 3). Moreover, 148 (13%) patients had taken platelet aggregation inhibitors and 58 (5%) had taken anticoagulation preoperatively. In addition, 575 (50%) patients had previously undergone abdominal and/ or urological surgery. 

RARP-treated patients with concomitant cardiovascular disease or concomitant chronic pulmonary disease were more likely to have short-term major postoperative complications (cardiovascular disease: 8 vs. 4%; *p* = 0.03; and chronic pulmonary disease: 13 vs. 5%; *p* = 0.02) compared to patients without the respective comorbidity (Table 3). For other comorbidities, no statistically significant differences were observed in the short-term major postoperative complications after RARP.

### 3.2. Univariable and Multivariable Logistic Regression Models

In the univariable logistic regression models, preoperatively diagnosed cardiovascular disease as well as preoperatively diagnosed chronic pulmonary disease were both associated with an increased risk of short-term major postoperative complications (cardiovascular disease: univariable odds ratio: 1.82; 95% confidence interval: 1.04–3.13; *p* = 0.03; and chronic pulmonary disease: univariable odds ratio: 2.93; 95% confidence interval: 1.16–6.40; *p* = 0.01; Table 4). After adjustment for other clinicopathological characteristics, concomitant cardiovascular disease and concomitant chronic pulmonary disease remained statistically significant risk factors for short-term major postoperative complications (Clavien Dindo ≥ IIIa) in multivariable logistic regression models (cardiovascular disease: multivariable odds ratio: 1.78; 95% confidence interval: 0.99–3.11; *p* = 0.046; and chronic pulmonary disease: multivariable odds ratio: 3.29; 95% confidence interval: 1.28–7.44; *p* = 0.007).

## 4. Discussion

Robotic-assisted radical prostatectomy has emerged as the standard of care for patients with localized prostate cancer [4,5]. Nonetheless, there remains a certain risk of postoperative complications that may not just impact patients’ well-being but also prolong inpatient stays [7,8]. Therefore, it is indispensable to analyze the rates of short-term major postoperative complications and to identify the risk factors for postoperative complications after RARP. Taking an aging society with increasing numbers of patients with comorbidities and complex medical conditions into account [12,15], we hypothesized that the short-term major postoperative complications after RARP do not differ significantly between prostate cancer patients with the most prevalent comorbidities, such as arterial hypertonia, elevated blood fat levels, diabetes mellitus, cardiovascular disease, chronic pulmonary disease, chronic kidney disease, other cancer, mental disorders, and neurologic disease, and their counterparts without the respective comorbidity. We tested this hypothesis on the most contemporary cohort of patients with localized prostate cancer who underwent RARP at a tertiary care referral center between January 2014 and March 2023 and made several noteworthy observations.

First, short-term major postoperative complications after RARP (Clavien Dindo ≥ IIIa) occurred in 5% of patients in the study cohort. Of those complications, 67% were classified as Clavien Dindo IIIa and hence manageable using procedures without anesthesia. No patient experienced a Clavien Dindo V postoperative complication. Moreover, 47% of the observed short-term major complications and the majority (61%) of postoperative complications classified as Clavien Dindo IIIa were caused by lymphoceles. Since there are no common guidelines regarding the interventions for lymphoceles, the institutional standard operating procedures (SOPs), including indication (e.g., pain, concomitant thrombosis, fever, elevated inflammatory parameters) as well as the approach to lymphocele treatment (e.g., percutaneous puncture, drainage, and operative fenestration), may vary significantly between different institutions. As a consequence, the overall major postoperative complications rates after radical prostatectomy are extremely variable between different institutions and ranged from 3% to 13% in previous studies [6,9,11,31,32]. In our series, complications were recorded prospectively in the electronic medical records of each patient, which might have led to a higher short-term major postoperative complication rate than in other comparable studies.

Second, we identified important differences in the baseline characteristics between patients with and without short-term major complications after RARP. In general, patients with short-term major postoperative complications after RARP (Clavien Dindo ≥ IIIa) more frequently exhibited high-risk prostate cancer (47 vs. 26%; *p* = 0.002), more frequently had lymph node invasion (20 vs. 6%; *p* < 0.001), and received more often neoadjuvant systemic therapy (17 vs. 5%; *p* = 0.002) compared to their counterparts without short-term major postoperative complications. These observations confirm the results of previously reported series [11,14]. Therefore, to reduce confounding or uncontrolled bias due to patient and tumor characteristics differences, it is crucial to adjust for these covariates in multivariable models, as was applied in the present study. 

Third, we assessed the distribution of common comorbidities among patients with localized prostate cancer who underwent RARP at our tertiary care referral center. Specifically, 486 (42%) patients were preoperatively diagnosed with concomitant arterial hypertonia, followed by 269 (23%) patients diagnosed with concomitant cardiovascular disease, and 237 (21%) patients diagnosed with concomitant obesity. As a consequence, arterial hypertonia, cardiovascular disease, and obesity represent the three most prevalent comorbidities in our study cohort of patients with localized prostate cancer who were treated with RARP. Furthermore, the currently recorded proportions of common chronic comorbidities among patients with prostate cancer are comparable with those observed in the general German population [25,33]. 

Fourth, patients with preoperatively diagnosed chronic pulmonary disease were more likely to have short-term major postoperative complications compared to patients without chronic pulmonary disease (13 vs. 5%; *p* = 0.02). In the multivariable logistic regression models, concomitant chronic pulmonary disease (odds ratio: 3.29; *p* = 0.007) remained independently associated with short-term major postoperative complications after RARP (Clavien Dindo ≥ IIIa). To the best of our knowledge, this is the first study that has investigated the impact of chronic pulmonary disease on the short-term major postoperative complications of patients with localized prostate cancer who underwent exclusively RARP. As a consequence, our findings cannot be directly compared with any previous study. However, these differences in short-term major postoperative complications are extremely worrisome and may be associated with prolonged postoperative recovery of lung function after use of the steep Trendelenburg position for RARP [34].

Fifth, patients preoperatively diagnosed with cardiovascular disease were more likely to have short-term major postoperative complications compared to patients without cardiovascular disease (8 vs. 4%; *p* = 0.03), too. Moreover, concomitant cardiovascular disease was independently associated with short-term major postoperative complications in the multivariable logistic regression models (odds ratio: 1.78; *p* = 0.046) while adjusting for other clinicopathological patient characteristics. The present observations, relying on a contemporary cohort of patients with localized prostate cancer who underwent RARP at our tertiary care referral center, do not only disprove our hypothesis but are also worrisome for both caregivers and patients. However, the currently recorded results are in agreement with historical series. Unfortunately, the previous series have solely examined specific cardiovascular conditions such as perioperative atrial fibrillation or prior percutaneous coronary intervention and have relied on heterogeneous cohorts of patients [12,18]. Taken together, cardiovascular disease and chronic pulmonary disease were both identified as independent risk factors for adverse short-term postoperative outcomes after radical prostatectomy. Therefore, both cardiovascular disease and chronic pulmonary disease should be taken into account during the preoperative medical assessment of patients with localized prostate cancer considered for RARP. 

Despite our noteworthy observations, the current study is not devoid of limitations. First, due to its retrospective nature and its limited sample size, a potential for residual selection biases, despite systematic adjustment for biases and confounders in the multivariable logistic regression models, remained. This limitation is applicable to all retrospective studies [10,22]. Second, the current analysis only addressed the most prevalent comorbidities. Due to the limited sample size and the limited number of events (only 60 patients with short-term major postoperative complications), specific concomitant diagnoses such as myocardial infarction and heart failure could not be analyzed individually and had to be grouped into categories, as defined in Supplementary Table S1. Moreover, rarer comorbidities, such as s history of prior organ transplantation and history of auto-immune disease, could not be assessed. Third, operative experience was variable among surgeons during the study period. To account for this observation, additional adjustment was made for surgeon volume in the multivariable logistic regression models. However, the surgeon volume was comparable between the two groups (with and without short-term major postoperative complications). Fourth, for the assessment of comorbidities, we relied on preoperative medical history interviews. Despite this structured data collection by both experienced anesthesiologists and urologists, objective examinations such as pulmonary function tests lead to higher estimates of prevalence [33]. Therefore, an underestimation of the prevalence of certain comorbidities and a selection bias cannot be excluded. Last but not least, the current study only examined the short-term major postoperative outcomes at 30 days follow-up. Consequently, no further comments can be made regarding minor postoperative complications classified as Clavien Dindo I or II, functional outcomes, such as incontinence and erectile function, or complications which occurred at a later stage [10,22]. Besides the very important primary endpoint of short-term major postoperative complications, upcoming studies should examine the role of concomitant comorbidities in equally important endpoints such as long-term complications, patients’ quality of life, and functional outcomes. 

## 5. Conclusions

In conclusion, short-term major postoperative complications after RARP only occurred in 5% of patients. These complications were largely caused by lymphoceles (47%), followed by bleeding-associated complications (13%), bowel-associated complications (8%), infections (5%), and fascia or wound dehiscence (5%). Moreover, the short-term major postoperative complications were predominantly classified as Clavien Dindo IIIa and therefore were manageable using procedures without anesthesia. No patient experienced a Clavien Dindo V postoperative complication. A history of arterial hypertonia, cardiovascular disease, obesity, other cancers, and diabetes mellitus represent the five most prevalent comorbidities among our cohort of patients with localized prostate cancer who underwent RARP at a tertiary care referral center. Concomitant cardiovascular disease and concomitant chronic pulmonary disease were both associated with a higher risk of short-term major postoperative complications after RARP (Clavien Dindo ≥ IIIa). As a consequence, concomitant cardiovascular disease and chronic pulmonary disease should be carefully considered during preoperative treatment decision-making by all clinicians taking care of patients with localized prostate cancer prior to RARP. 

## Figures and Tables

**Table 1:** Rates of short-term major postoperative complications after robotic-assisted radical prostatectomy (RARP) according to Clavien Dindo classification system.

Characteristic	Clavien Dindo ≥ IIIa, 60 (100% ^1^; 5% ^2^)	Clavien Dindo IIIa 38 (63% ^1^; 3.3% ^2^)	Clavien Dindo IIIb 16 (27% ^1^; 1.4% ^2^)	Clavien Dindo IVa 4 (7% ^1^; 0.3% ^2^)	Clavien Dindo IVb 2 (3% ^1^; 0.2% ^2^)
Lymphocele	28 (47%) ^1^	23 (61%) ^3^	5 (31%) ^3^	0 (0%) ^3^	0 (0%) ^3^
Infection	3 (5%) ^1^	1 (3%) ^3^	2 (13%) ^3^	0 (0%) ^3^	0 (0%) ^3^
Bleeding, hematoma, tamponade	8 (13%) ^1^	4 (11%) ^3^	3 (19%) ^3^	1 (25%) ^3^	0 (0%) ^3^
Thrombosis, embolism	1 (2%) ^1^	0 (0%) ^3^	0 (0%) ^3^	1 (25%) ^3^	0 (0%) ^3^
Fascia or wound dehiscence	3 (5%) ^1^	2 (5%) ^3^	1 (6%) ^3^	0 (0%) ^3^	0 (0%) ^3^
Cardiac complications	2 (3%) ^1^	1 (3%) ^3^	0 (0%) ^3^	1 (25%) ^3^	0 (0%) ^3^
Bowel damage, ileus	5 (8%) ^1^	0 (0%) ^3^	2 (13%) ^3^	1 (25%) ^3^	2 (100%) ^3^
Other complications	10 (17%) ^1^	7 (17%) ^3^	3 (18%) ^3^	0 (0%) ^3^	0 (0%) ^3^

^1^ % of Clavien Dindo ≥ IIIa complications; ^2^ % of all patients; ^3^ % of Clavien Dindo IIIa, IIIb, IVa, or IVb complications.

**Table 2:** Descriptive characteristics of prostate cancer patients undergoing robotic-assisted radical prostatectomy (RARP) between January 2014 and March 2023.

Characteristic	Overall, n = 1148 ^1^	Clavien Dindo ≥ IIIa, n = 60 (5%) ^1^	No Clavien Dindo ≥ IIIa, n = 1088 (95%) ^1^	*p*-Value ^2^
Age (in years)	65 (60, 70)	67 (62, 71)	65 (60, 70)	0.3
D’Amico risk group				0.002
Low	181 (16%)	5 (8%)	176 (16%)	
Intermediate	649 (57%)	27 (45%)	622 (58%)	
High	311 (27%)	28 (47%)	283 (26%)	
Gleason sum score biopsy				0.02
6	262 (23%)	9 (15%)	253 (23%)	
7	657 (57%)	31 (52%)	626 (58%)	
≥8	227 (20%)	20 (33%)	207 (19%)	
cTstage				0.2
≤cT2a	922 (80%)	45 (75%)	877 (81%)	
cT2b	90 (8%)	3 (4%)	87 (8%)	
≥cT2c	126 (11%)	12 (20%)	114 (10%)	
Unknown	10 (1%)	0 (0%)	10 (1%)	
PSA at diagnosis (in ng/mL)	7.1 (5.2, 10.9)	9.7 (6.4, 16.9)	7.0 (5.2, 10.6)	0.002
<10	794 (71%)	31 (53%)	763 (71%)	0.007
10–20	225 (20%)	16 (28%)	209 (20%)	
>20	107 (9%)	11 (19%)	96 (9%)	
Neoadjuvant systemic therapy	67 (6%)	10 (17%)	57 (5%)	0.002
Surgeon volume	69 (29, 169)	62 (33, 163)	70 (29, 170)	0.9
Operation time (in min)	188 (156, 239)	189 (160, 256)	188 (156, 238)	0.7
FFLU + NeuroSAFE	989 (86%)	941 (86%)	48 (80%)	0.2
Pelvic lymph node dissection	1015 (88%)	58 (97%)	957 (88%)	0.04
pNstage				<0.001
N0	948 (83%)	46 (79%)	902 (83%)	
N1	74 (6%)	12 (20%)	62 (6%)	
NX	126 (11%)	2 (3%)	124 (11%)	

Abbreviations: cTstage = clinical tumor stage; FFLU = full functional-length urethral sphincter preservation; NeuroSAFE = neurovascular structure-adjacent frozen-section examination; pNstage = pathological lymph node stage; PSA = prostate-specific antigen. ^1^ Median (IQR); n (%); ^2^ Wilcoxon rank sum test; Pearson’s Chi-square test; Fisher’s exact test.

**Table 3:** Prevalence of comorbidities and associated rates of short-term major postoperative complications after robotic-assisted radical prostatectomy (RARP).

Characteristic	Prevalence ^1^	Clavien Dindo ≥ IIIa in Patients with the Respective Comorbidity ^1^	Clavien Dindo ≥ IIIa in Patients without the Respective Comorbidity ^1^	*p*-Value ^2^
Arterial hypertonia	486 (42%)	24 (4.9%)	36 (5.4%)	0.7
Elevated blood fat levels	64 (6%)	4 (6.3%)	56 (5.2%)	0.6
Obesity (BMI ≥ 30)	237 (21%)	16 (6.8%)	44 (4.8%)	0.2
Diabetes mellitus	105 (9%)	8 (7.6%)	52 (5.0%)	0.2
Cardiovascular disease	269 (23%)	21 (7.8%)	39 (4.4%)	0.03
Platelet aggregation inhibitor	148 (13%)	8 (5.4%)	52 (5.2%)	0.9
Anticoagulation	58 (5%)	4 (6.9%)	56 (5.1%)	0.5
Chronic pulmonary disease	54 (5%)	7 (13.0%)	53 (4.8%)	0.02
Chronic kidney disease	32 (3%)	1 (3.1%)	59 (5.3%)	0.9
Other cancer	107 (9%)	8 (7.5%)	52 (5.0%)	0.3
Mental disorders	31 (3%)	2 (6.5%)	58 (5.2%)	0.7
Neurological disease	100 (9%)	4 (4.0%)	56 (5.3%)	0.6
Previous urological and/or abdominal surgery	575 (50%)	31 (5.4%)	29 (5.1%)	0.8

Abbreviations: BMI = body mass index. ^1^ n (%); ^2^ Wilcoxon rank sum test; Pearson’s Chi-square test; Fisher’s exact test.

**Table 4:** Univariable and multivariable logistic regression models (LRMs) addressing short-term major postoperative complications after robotic-assisted radical prostatectomy (RARP).

	Univariable			Multivariable *		
	OR	95% CI	*p*-Value	OR	95% CI	*p*-Value
Cardiovascular disease (Ref. no)	1.82	1.04, 3.13	0.03	1.78	0.99, 3.11	0.046
Chronic pulmonary disease (Ref. no)	2.93	1.16, 6.40	0.01	3.29	1.28, 7.44	0.007

Abbreviations: CI = confidence interval; FFLU = full functional-length urethral sphincter preservation; NeuroSAFE = neurovascular structure-adjacent frozen-section examination; OR = odds ratio; pNstage = pathological lymph node stage. * Adjusted for D’Amico risk group, neoadjuvant systemic therapy, surgeon volume, surgical technique (FFLU + NeuroSAFE) vs. standard), pNstage, cardiovascular disease, chronic pulmonary disease.

## Data Availability

All data generated or analyzed during this study are included in this article. Further enquiries can be directed to the corresponding author.

## Supplementary Materials

The following supporting information can be downloaded at https://www.mdpi.com/article/10.3390/medicina60010173/s1. Table S1: Detailed description of comorbidities.
