# Supplementary material for: Cardiovascular Disease and Chronic Pulmonary Disease Increase the Risk of Short-Term Major Postoperative Complications after Robotic-Assisted Radical Prostatectomy
Source: Medicina (Kaunas). 2024 Jan 18;60(1):173. doi: 10.3390/medicina60010173 (PMC10820446; doi:10.3390/medicina60010173)
Supplement: Supplementary file 1 [file medicina-60-00173-s001.zip › medicina-2794514-supplementary.pdf]

Comorbidity	Detailed desricption
**Arterial hypertonia**	Arterial hypertonia, hypertensive heart disease.
**Elevated blood fat levels**	Hypercholesterolemia, hyperlipoproteinemia, hyperlipidemia, dyslipidemia.
**Obesity**	Body mass index (BMI) ≥ 30kg/m^2.
**Diabetes mellitus**	Diabetes mellitus type 1 and 2.
**Cardiovascular disease**	myocardial infarction, coronary heart disease, heart failure, left ventricular hypertrophy, thrombosis, mitral regurgitation, carotis-TEA, atrio-ventricular-re-entry tachycardia, aneurysm, thrombosis of the portal vein, heart-valve surgery, tachyarrhythmia, aortic valve insufficiency, tricuspid insufficiency, Churg-Strauss syndrome, atrial fibrillation, left bundle branch block, deep vein thrombosis, pacemaker implantation, bradycardia syndrome, SVES, extrasystole, stenosis of the aortic isthmus, right bundle branch block, mitral valve repair, annuloplasty, aortocoronary bypass, aortic valve replacement, ministernotomy, hypertensive heart disease, cardioversion, heart bypass, carotid stenosis, tendency to collapse, dissection, myocarditis, lungs artery embolism, macroangiopathy, carotid sclerosis, heart valve insufficiency, arrhythmia, microangiopathy, anal venous thrombosis, persistent foramen ovale, Hypertrophic cardiomyopathy, dilatative cardiomyopathy, catheter ablation, mitral valve stenosis, ocular vein thrombosis, sinus bradycardia, ventricular tachycardia, ventricular fibrillation, tachycardia, aortoarteriopathy, aortic aneurysm, Henoch–Schönlein purpura, thrombophlebitis, endocarditis, aortic valve sclerosis, renal artery stenosis, peripheral arterial occlusive disease, arteriosclerosis, atherosclerosis, catheter ablation of atrial fibrillation, atrium resection, stent angioplasty, aortic valve stenosis, pelvic vein thrombosis, inferior vena cava filter, atrioventricular block, aortic ectasia, syncope, pulmonary vein isolation, Paget-von-Schroetter-syndrome, tricuspid valve reconstruction, cryo coronary ablation, defibrillation, automatic implanted cardiac defibrillator, cardiomyopathy, palpitation, heart surgery, DDD-pacemaker, aterio-ventricular knot ablation, aortic valve reconstruction, occluder, thrombectomy, embolism, ventricular septum defect, atrial septum defect, sick-sinus-syndrome, retina thrombosis, asystole, central vein thrombosis, EVAR, intermittent claudication, myocarditis, drug-eluting stent, pericardial effusion, Y-Prothesis, Wolff-Parkinson-White syndrome, stent implantation, medtronic, coronary sclerosis, heart-valve replacement, aneurysm rupture, arterial-venous bypass, embolic infarctions.
**Platelet aggregation inhibitor**	ASS, Clopidogrel, Prasugrel, Ticlopidin, Ticagrelor.

**Anticoagulation**	Dabigatran, Pradaxa, Rivaroxaban, Xarelto, Apixaban, Eliquis, Edoxaban, Lixiana, Marcumar, Cumarine.
**Chronic pulmonary disease**	Chronic obstructive pulmonary disease, obstructive bronchitis, bronchial asthma, hyper-responsive bronchial system, pulmonary emphysema, pulmonary fibrosis, pulmonary sarcoidosis.
**Chronic kidney disease**	Renal insufficiency, renal failure, single kidney, functionless kidney, nephrectomy, kidney transplant.
**Other cancer**	Bladder cancer, urothelial carcinoma, melanoma, thyroid carcinoma, basal cell carcinoma, germ cell tumour, breast cancer, renal cell carcinoma, testicle cancer, seminoma, folfox, chronic myeloid leukemia, stem cell transplant, embryonal teratoma, hepatocellular carcinoma, liposarcoma, adeno carcinoma, polycythemia, carcinoma, cancer, myeloproliferative neoplasm, skin cancer, colon cancer, tongue carcinoma, laryngeal carcinoma, gastric cancer, chemotherapy, radiochemotherapy, lung cancer, chronic lymphocytic leukemia, Bowen's disease,<br>CUP syndrome, Waldenström macroglobulinemia, non-Hodgkin's lymphoma, rectal cancer, spino-cellular carcinoma, giant cell carcinoma, leukemia, tumor, neuroendocrine tumor, esophageal cancer, mixed tumor, myelodysplastic syndrome, colorectal cancer, CHOEP, rhabdomyosarcoma, hypo-pharyngeal carcinoma, gastrointestinal stromal tumor, acute myeloid leukemia,<br>parotid gland tumor, leydig-cell-tumor, myeloma,<br>sigma carcinoma, pseudomyxomia.
**Mental disorder**	Anxiety disorder, depression, schizo-effective disorder, panic disorder, attention-deficit/hyperactivity syndrome, chronic pain disorder, burnout.
**Neurological disease**	Parkinson's disease, subdural hematoma, basilar skull fracture, tension headache, migraine, numbness, Guillain-Barré syndrome, subdural empyema, carpal tunnel syndrome, stiffness, hypoglossal paresis, hemiplegia, neurinoma, postdisectomy-syndome, restless legs syndrome, radiculopathy, hemiparesis, meningioma, ophthalmoplegia, vocal cord paralysis, paralysis of the recurrent nerve, neuropathy, polyneuropathy, myelitis, paraplegia, coma, sensomotoric lesion of the Plexus brachialis, craniocerebral trauma, epilepsy, lower limb paralysis, poliomyelitis, meningitis, cavernoma, glossopharyngeal neuralgia, paraplegic myelitis, paraspastic, neurolues, paraparesis, schwannoma, Alzheimer's disease, acoustic neurinoma, tremor, cerebral concussion, intracerebral hemorrhage, cerebellar meningioma, trigeminal neuralgia, foot flexor weaknesses, autoimmune encephalitis, vestibularis schwannoma, hemianopsia, facial nerve paresis, multiple sclerosis, stroke.
